# Interrelationship between type three secretion system and metabolism in pathogenic bacteria

**DOI:** 10.3389/fcimb.2014.00150

**Published:** 2014-10-27

**Authors:** Gottfried Wilharm, Christine Heider

**Affiliations:** Project Group P2, Robert Koch-InstituteWernigerode, Germany

**Keywords:** virulence, metabolism, type three secretion system, T3SS, cross-talk, *Yersinia*, *Salmonella*, *Pseudomonas*

## Abstract

Before the advent of molecular biology methods, studies of pathogens were dominated by analyses of their metabolism. Development of molecular biology techniques then enabled the identification and functional characterisation of the fascinating toolbox of virulence factors. Increasing, genomic and proteomic approaches form the basis for a more systemic view on pathogens' functions in the context of infection. Re-emerging interest in the metabolism of pathogens and hosts further expands our view of infections. There is increasing evidence that virulence functions and metabolism of pathogens are extremely intertwined. Type three secretion systems (T3SSs) are major virulence determinants of many Gram-negative pathogens and it is the objective of this review to illustrate the intertwined relationship between T3SSs and the metabolism of the pathogens deploying them.

## Introduction

In the introduction to a review on “Metabolism of Microorganisms as related to their pathogenicity” published more than 50 years ago, Panos and Ajl stated, “The problem of how certain aspects of the metabolism of a bacterial cell are related to its pathogenicity is an exceedingly complex and difficult one” (Panos and Ajl, 1963). As will be outlined here, our present view on this subject could hardly be formulated more to the point. Elsewhere in their review, Panos and Ajl state that “This review is not intended to be an exhaustive coverage of the voluminous literature available on this subject,” indicating that many forgotten treasures could be raised from that past literature, including important details that newcomers to the field might not know (Panos and Ajl, 1963). Indeed, as will be also illustrated here, many of these early studies can now be linked to more recent ones, mounting increasingly complex regulatory networks in which functions of metabolism and virulence are closely intertwined. Other early studies might serve as stimuli for reinvestigation using state-of-the-art methodology.

It is obvious that a pathogen requires metabolic functions in order to establish an infection and that these metabolic functions adapt to nutritional situation(s) during infection. Consequently, the metabolism of pathogens during infection can differ substantially from that of the *in vitro* situation (Munoz-Elias and McKinney, 2006). It is also known that sensing specific nutrients together with other environmental conditions that are indicative of a host environment stimulates production of virulence factors in many pathogens (Poncet et al., 2009). Further, global regulatory networks such as the stringent response system, which responds to various nutritional and metabolic stresses, or the carbon catabolite repression and carbon storage regulator systems, respectively, play an important role in the control of virulence traits (Romeo, 1998; Gorke and Stulke, 2008; La et al., 2008; Poncet et al., 2009; Dalebroux et al., 2010; Heroven et al., 2012). Though important, these aspects are not the focus of this review, are treated elsewhere and will only be mentioned briefly. The question we address is whether there is anything beyond this global and well-established relationship in the interrelatedness of virulence functions and metabolism in bacterial pathogens.

It will become apparent in this review that the link between virulence and metabolism is so important in many pathogens that we are probably only seeing the tip of the iceberg. Using the example of the type three secretion system (T3SS), a prototypic virulence determinant of many Gram-negatives, we will show in particular that not only metabolism controls and underpins production and functioning of virulence factors but also that, conversely, virulence factors can modulate metabolic functions of pathogens in a coordinate way, as for example, in *Yersinia* (Moncla et al., 1983; Du et al., 2009; Schmid et al., 2009) or pave the way for novel metabolic options as in *Salmonella* (Winter et al., 2010) and *Pseudomonas* (Dacheux et al., 2001b). The following criteria served as indicators to identify similar phenomena in other pathogens: (i) Can it be assumed or is it known that deletion of a T3SS component results in changes of metabolism? (ii) Is there evidence that such metabolic changes are due to a specific interference between T3SS and metabolic functions rather than due to global metabolic effects (e.g., relief from a global metabolic burden due to the loss of a virulence plasmid). (iii) Assuming global effects in general, studies of specific metabolic functions required for full virulence will not been considered unless the metabolic functions can be linked to specific T3SS components (e.g., deletion of a metabolic function specifically interferes with T3SS gene expression). Examples of interrelatedness between T3SS functions and metabolism will be followed by discussion of common principles and their impact on our view of bacterial pathogenesis.

### *Yersinia*: cross-talk between a type three secretion system and metabolism

Studies linking metabolism and virulence in *Yersinia pestis* (originally called *Pasteurella pestis*), the causative agent of plague, go back more than 50 years. After Devignat and Schoetter (1942) reported in 1942 that virulent strains of *Y. pestis* become avirulent upon continued aerobic culturing at 37°C, Fukui et al. (1957) showed that loss of virulence was accompanied by a loss of the so-called V-antigen (Bacon and Burrows, 1956). Only decades later did it become clear that V-antigen (LcrV) was a key component of the plasmid-encoded *Yersinia* type three secretion system (T3SS), a major virulence determinant shared by *Y. pestis* and the enteropathogenic species *Y. pseudotuberculosis* and *Y. enterocolitica*. It was this virulence plasmid, termed pCD in *Y. pestis* and pYV in *Y. pseudotuberculosis* and *Y. enterocolitica*, that was rapidly lost at 37°C *in vitro* (Cornelis et al., 1998). Higuchi and Carlin (Higuchi and Carlin, 1958) found that virulent strains grew faster than avirulent types at 37°C but not at 27°C. A search for media promoting growth of virulent strains of *Y. pestis* revealed a critical role of calcium ions preventing the emergence of avirulent variants (Kupferberg and Higuchi, 1958). Shortly after, Delwiche et al. (1959) noted that bicarbonate, aspartate and glutamate also prevented loss of virulence. How can these early observations be interpreted in the light of the current knowledge and do they point to a specific interrelationship between virulence and metabolism in *Yersinia*? First, production and assembly of the *Yersinia* T3SS machinery occur only at 37°C (Yother et al., 1986), and a low concentration of calcium ions triggers the massive secretion of T3SS substrate proteins called Yops into the culture supernatant (Heesemann et al., 1984). It seemed reasonable to assume that the poor growth of pathogenic *Yersinia* at 37°C under low calcium conditions could be explained by the heavy metabolic burden imposed by the massive production and secretion of Yops (Ramamurthi and Schneewind, 2002). This interpretation was supported by the fact that loss of the virulence plasmid encoding the T3SS relieves from this burden but the effect of bicarbonate, aspartate and glutamate could not be explained.

The fact that bicarbonate prevented the loss of virulence in *Y. pestis* (Delwiche et al., 1959) stimulated Baugh et al. (1964) to compare carbon dioxide fixation reactions in extracts from virulent and avirulent strains. Although Baugh et al. did not observe any significant difference (Baugh et al., 1964), it seems now likely that their hypothesis was correct, since there is a direct linkage between carbon dioxide fixation and T3SS in *Yersinia* (Schmid et al., 2009). Specifically, two regulatory components of the *Y. enterocolitica* T3SS, YscM1 and YscM2, bind directly to the phosphoenolpyruvate carboxylase (PEPC) of *Yersinia*. PEPC catalyses the carboxylation of phosphoenolpyruvate (PEP) to form oxaloacetate, a replenishing or so-called anaplerotic reaction. The PEPC reaction replenishes the tricarboxylic acid (TCA) cycle that, besides generating reduction equivalents, provides building blocks for amino acid synthesis. More precisely, oxaloacetate is used to form aspartate and other amino acids of the aspartate family, while α-ketoglutarate is used to form glutamate and related amino acids. It is important to remember that aspartate and glutamate both suppress growth restriction in *Y. pestis* (Delwiche et al., 1959), and that activation of the *Yersinia* T3SS requires glutamate, glutamine, aspartate, or asparagine (Lee et al., 2001). Furthermore, alkaline pH, which favors formation of bicarbonate from CO_2_, as well as bicarbonate, mitigates growth restriction of pathogenic *Yersinia* (Ogg et al., 1958; Delwiche et al., 1959). So, is the interaction between the T3SS regulators YscM1/YscM2 and the CO_2_-fixing PEPC the key to understanding *Yersinia* growth cessation under conditions leading to T3SS activation?

The *Yersinia* virulence plasmid-encoded T3SS comprises about 45 components (Cornelis et al., 1998). The regulatory gene *lcrQ* in *Y. pestis* and *Y. pseudotuberculosis* was presumably duplicated in *Y. enterocolitica* to give *yscM1*, encoding a protein 99% identical to LcrQ, and, *yscM2*, whose product is almost 60% identical to YscM1 and LcrQ. YscM1 and YscM2 are functionally redundant in the control of *yop* gene expression (Stainier et al., 1997). However, while both were shown to interact with PEPC, only YscM1 influenced PEPC activity *in vitro* (Schmid et al., 2009). In fact, YscM1 is a potent inhibitor of PEPC. What is more, YscM1 (LcrQ) and YscM2 are also T3SS secretion substrates and are injected into host cells (Pettersson et al., 1996; Cambronne et al., 2000). Their status as secretion substrates is crucial since induction of type III secretion (accompanied by growth cessation) decreases intracellular YscM/LcrQ levels (Pettersson et al., 1996). In other words, how can inhibition of PEPC activity by YscM1/LcrQ explain growth restriction given that the YscM1/LcrQ level in *Yersinia* is low under these conditions? Perhaps this has to do with the complex protein/protein interaction network in which these regulators are intertwined with at least seven possible interaction partners besides PEPC (Cambronne et al., 2000; Swietnicki et al., 2004; Dittmann et al., 2007; Wilharm et al., 2007; Li et al., 2014a,b). Moreover, there is also evidence that PEPC is not the only junction between the YscM proteins and metabolism in *Yersinia*.

Stable isotope labeling experiments with universally ^13^C-labeled glucose followed by mass spectrometry analyses of the labeling patterns of amino acids were performed to assess the influence of PEPC, YscM1 and YscM2 on carbon fluxes during Yop secretion (Schmid et al., 2009). The data showed that PEPC replenishes the oxaloacetate pool of the TCA cycle under Yop secretion conditions. Deletion of either *yscM1* or *yscM2* caused slight changes of carbon fluxes in glycolysis and/or the Entner-Doudoroff pathway, the pentose phosphate pathway, the TCA cycle and amino acid biosynthesis. Collectively, these results suggest that not only the PEPC reaction but also one or several other central metabolic reactions are modulated via *yscM1/yscM2*. Strikingly, while purified YscM1 and YscM2 behaved differently with respect to PEPC inhibition *in vitro*, as mentioned above, the *yscM1* and *yscM2* deletion strains were indistinguishable with respect to ^13^C labeling patterns.

Although it is evident that much has to be done to get a clearer picture of the interrelatedness of virulence and metabolism in *Yersinia*, it is already reasonable to speculate on possible functions of such a cross-talk. *Yersinia* type III secretion is demanding with respect to both synthesis of Yops and provision of energy for their secretion (Wilharm et al., 2007; Schmid et al., 2009). Thus, it seems plausible that functioning of the T3SS under *in vivo* conditions requires the coordinated adaptation of metabolic pathways. Due to their bifunctionality, YscM/LcrQ proteins are ideally suited to coordinate *yop* gene expression and metabolic requirements. Since YscM/LcrQ interact with multiple T3SS components, and in particular chaperones of the Yop effectors, they might integrate information on the status of the T3SS, e.g., by sensing if the chaperones are charged with Yops, and transduce this information into control of both Yop production and the anaplerotic PEPC reaction, thereby balancing amino acid biosynthesis and energy provision.

A crucial role of the *Yersinia* T3SS is to prevent phagocytosis. The T3SS is therefore fully assembled at 37°C and a pre-synthesized pool of Yops is “ready-to-go” for microinjection. As the extracellular milieu of the host is rich in calcium, growth restriction will not occur and *Yersinia* is able to replicate. Upon phagocytic attack, a very rapid and concerted reaction is pivotal for *Yersinia*. It seems plausible that maintenance of energy charge for translocation of Yop effectors and replenishment of Yops have to be prioritized over continued replication under these conditions. Sensing an attacking cell might thus trigger a growth cessation programe, accompanied by reprogramming of metabolic pathways in which the PEPC-YscM/LcrQ interaction plays a role.

In support, Meng et al. (2010) demonstrated that phosphoenolpyruvate synthase and glutaminase change phosphorylation/modification status upon induction of the T3SS in *Y. enterocolitica*.

### Aspartase: a key to understanding *Yersinia* virulence

Fully virulent epidemic isolates of *Y. pestis* exhibit a lower aspartase activity than attenuated *Y. pestis* isolates and the enteropathogenic *Y. pseudotuberculosis* and *Y. enterocolitica* (Dreyfus and Brubaker, 1978; Bearden et al., 2009; Bearden and Brubaker, 2010). Aspartase (aspartate ammonia lyase, AspA) catalyses the deamination of aspartate to yield fumarate, an intermediate of the tricarboxylic acid (TCA) cycle. This reaction can thus be regarded as anaplerotic, and its absence from *Y. pestis* results in a net loss of carbon excreted in the form of aspartate (Dreyfus and Brubaker, 1978; Bearden and Brubaker, 2010). Fowler and Brubaker (1994) pointed out a specific role of carbon dioxide fixation reactions in relation to the aspartase deficiency identified in *Y. pestis* (Dreyfus and Brubaker, 1978). Carbon dioxide fixing reactions such as formation of the TCA-cycle intermediate oxaloacetate from phosphoenolpyruvate by the phosphoenolpyruvate carboxylase (PEPC, see previous section) might be especially important to replenish the TCA cycle and to compensate for the aspartase deficiency in *Y. pestis*. The aspartase deficiency might explain why growth restriction associated with low calcium response (LCR) is more prominent in *Y. pestis* compared to the enteropathogenic *Yersinia* (Bearden and Brubaker, 2010). According to Brubaker and colleagues, a key to explaining the exceptional virulence of epidemic *Y. pestis* compared to attenuated enzootic strains and to the enteropathogenic *Yersinia* is to understand how aspartase deficiency increases the virulence of *Y. pestis* (Viola et al., 2008; Bearden et al., 2009; Bearden and Brubaker, 2010). In support of a direct linkage between virulence and aspartase in *Y. pestis, aspA* is down-regulated in a mutant strain lacking the T3SS regulator gene *lcrG* (Du et al., 2009).

### Uptake and degradation of fatty acids depends on the presence of virulence plasmids in *Y. pestis*

The cross-talk between virulence functions and metabolism in *Y. pestis* is presumably even more complicated. Moncla et al. (1983) demonstrated constitutive uptake and degradation of fatty acids in *Y. pestis* via β–oxidation and the glyoxylate shunt. Intriguingly, fatty acid uptake was found to depend on the T3SS-encoding plasmid pCD1 as well as on the small virulence plasmid encoding the plasminogen activator (Pla) and pesticin (Pst). Unfortunately, these interesting findings were not followed-up. However, transcriptional profiling of wild-type *Y. pestis* and a strongly attenuated mutant lacking *lcrG* indicated up-regulation of *aceA* and *aceB*, encoding the key enzymes of the glyoxylate shunt, isocitrate lyase and malate synthase, in the *lcrG* mutant, which supports the interrelationship between T3SS and fatty acid metabolism (Du et al., 2009). Furthermore, differential expression of *aceA* and *aceB* upon a shift from 26 to 37°C was reported in two studies (Han et al., 2004; Motin et al., 2004), supporting a specific role of fatty acid metabolism during infection.

Another illustration of the complexity of the network is the fact that acetyl-CoA, fatty acids and fatty acyl-CoA are allosteric activators of PEPC, and β–oxidation of fatty acids, which leads to acetyl-CoA, requires oxaloacetate as an acceptor (Morikawa et al., 1980; Sauer and Eikmanns, 2005). Furthermore, L-malate, a product of the glyoxylate shunt reactions, is an allosteric inhibitor of PEPC.

### Catabolite repression controls type III effector production in *Yersinia*

Zhan et al. (2008, 2009) demonstrated that cyclic AMP receptor protein (CRP) controls expression of the *sycO*-*ypkA*-*yopJ* operon in *Y. pestis*. This operon encodes two effectors of the T3SS, YopJ (YopP in *Y. enterocolitica*) and YpkA (YopO in *Y. enterocolitica*), together with SycO, the YpkA chaperone. The cAMP/CRP complex, but not CRP alone, binds to the promoter region of *sycO*-*ypkA*-*yopJ*, suggesting that high cAMP levels repress the *sycO*-*ypkA*-*yopJ* operon (Zhan et al., 2009). Zhan et al. (2008) defined a minimal CRP regulon by identifying all genes affected by the *crp* deletion. Besides *ypkA* the 37 genes or operons of the minimal CRP regulon include those encoding pesticin (*pst*) and plasminogen activator (*pla*), which are on the small virulence plasmid. Strikingly, expression of aspartase gene *aspA* was also strongly influenced by *crp* (Zhan et al., 2008). While *Δcrp* substantially increased *ypkA* expression, it dramatically lowered expression of *pst, pla* and *aspA*, suggesting these inversely regulated factors are required under completely different physiological conditions. These results indicate the effector YpkA (and possibly also YopJ) is only engaged under certain physiological conditions, since other *yop* genes are not part of the CRP regulon (Zhan et al., 2008). It is also worth mentioning that SycO interacts with YscM1 and regulates Yop production (Dittmann et al., 2007). SycO may thus also interfere with the PEPC-YscM1 regulatory network.

**Figure 1:**
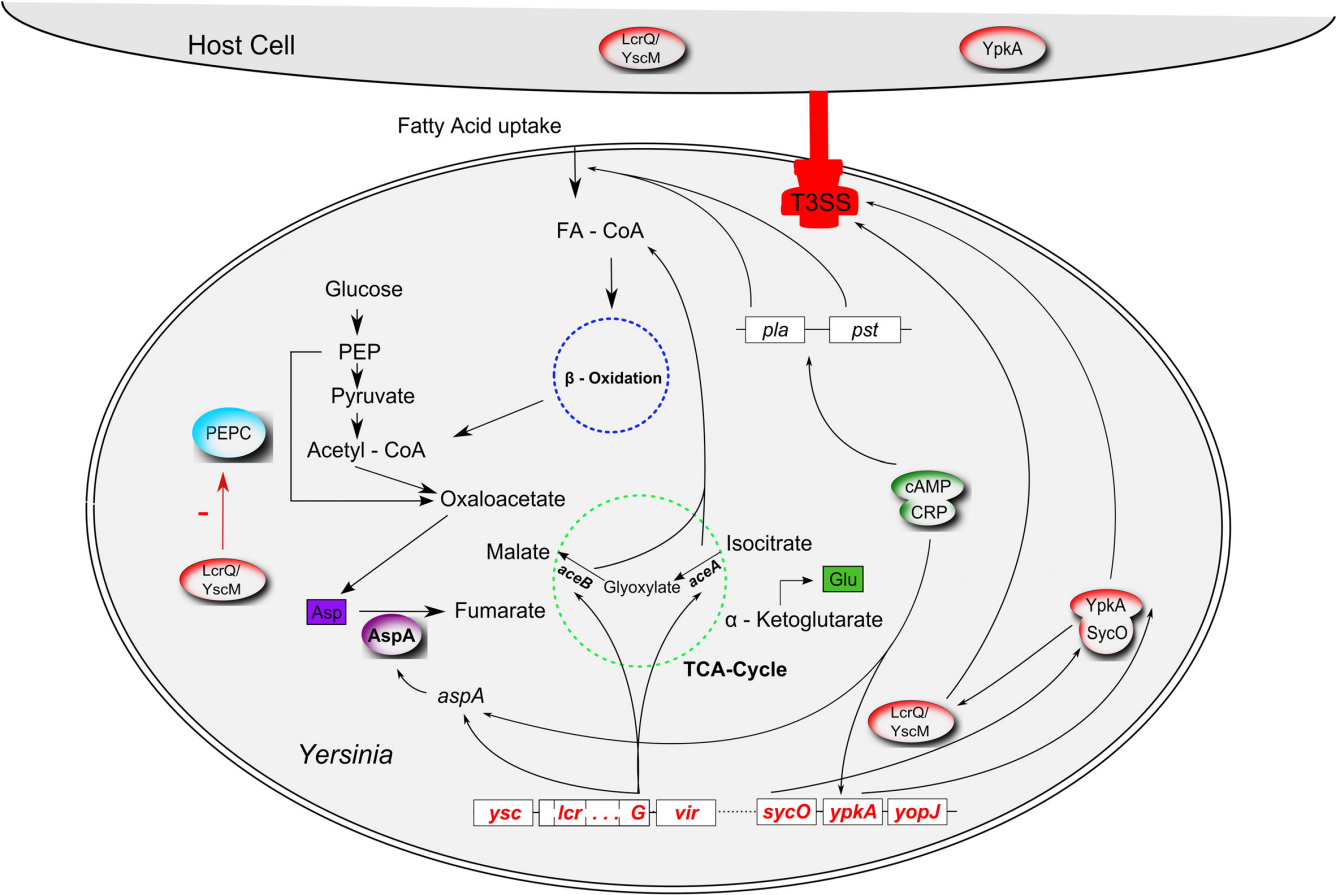
**Interrelations between virulence factors and metabolism in *Yersinia***. The type 3 secretion system (T3SS; red), encoded by the pCD virulence plasmid (called pYV in *Y. enterocolitica*) interferes with the anaplerotic enzyme phosphoenolpyruvate carboxylase (PEPC, blue) via the T3SS regulator LcrQ (YscM1 in *Y. enterocolitica*; inhibitor of PEPC); *lcrG*, another component of the low calcium response regulon, controls transcription of *aceAB* and *aspA*. The glyoxylate cycle enzymes isocitrate lyase and malate synthase, encoded by *aceAB*, fulfill an anaplerotic function required for fatty acid degradation (FA, fatty acid; FA-CoA, fatty acyl-CoA). AspA, an aspartase generating fumarate from aspartate can also be regarded as an anaplerotic enzyme. AspA seems to be catalytically inactive in *Y. pestis*. In an unknown way, plasmid pCD seems to be involved in fatty acid uptake together with another virulence plasmid, pPCP. Production of the pPCP-encoded virulence factors Pla (plasminogen activator) and Pst (pesticin) is controlled by cAMP (cyclic adenosine monophosphate) via the cAMP receptor protein CRP (“catabolite repression”). The cAMP/CRP complex represses the T3SS effector gene *ypkA* (*yopO* in *Y. enterocolitica*). SycO, the chaperone of YpkA, directly interacts with LcrQ/YscM1. Cross-talk between fatty acid metabolism, glyoxylate shunt and the PEPC reaction also occurs via allosteric effectors (FA, FA-CoA, malate; interactions not illustrated for reasons of clarity).

Taken together, a densely interwoven regulatory network connects the T3SS along with other virulence factors to the central carbon metabolism in *Yersinia*. The relationships depicted above are graphically summarized in Figure 1. It is important to note that very recently a study on the evolution of *Yersinia* identified that different from the previous view (Carniel, 2002; Wren, 2003) acquisition of the pYV virulence plasmid occurred independently in the *Y. enterocolitica* and *Y. pseudotuberculosis*/*Y. pestis* lineages after their separation (Reuter et al., 2014). Consequently, the regulatory networks in which the T3SSs are embedded can differ for the two lineages as can the specific interrelations between metabolism and the respective T3SS.

### *Pseudomonas aeruginosa* T3SS: a hunting weapon?

The opportunistic pathogen *Pseudomonas aeruginosa* deploys a T3SS with many constituents highly homologous to that of the *Yersinia* T3SS. Their regulatory networks differ considerably, however, because *Pseudomonas* lacks a homolog of the *Yersinia* LcrQ/YscM regulators. Another important difference concerns the use of the respective T3SS. While immune evasion and avoidance of collateral damage seems to be a major issue for *Yersinia*, the situation seems less clear for *P. aeruginosa*. The pore-forming activity of the *P. aeruginosa* T3SS results in macrophage oncosis and release of a chemoattractant from these oncotic cells (Dacheux et al., 2001b). This leads to rapid accumulation of pseudomonads around the cells, a phenomenon called pack swarming. It seems plausible that leakage of the oncotic cells is exploited by the bacteria for their nutrition and, thus, that the *Pseudomonas* T3SS is deployed both for immune evasion and as a weapon to hunt for nutrients.

Several studies have shown that production of the T3SS of *Pseudomonas aeruginosa* is affected by the metabolic state of the cells. The pyruvate dehydrogenase operon *aceAB* (please note the confusing nomenclature, these genes are unrelated to genes with identical names encoding the isocitrate lyase and malate synthase enzymes of the glyoxylate shunt) is required for maximal production of the T3SS (Dacheux et al., 2002). While the growth defect of *aceAB* mutants could be restored by acetate supplementation, the associated defect in T3SS production could be not, suggesting an interaction between *aceAB* gene products and the T3SS signaling cascade, rather than a global metabolic defect restricting expression of the T3SS genes.

Under T3SS-inducing conditions, adenylate cyclases CyaA and CyaB control expression of the T3SS genes via the cAMP binding protein Vfr (Wolfgang et al., 2003; Smith et al., 2004; Rietsch and Mekalanos, 2006), but global catabolite repression control in *Pseudomonas* is not critically dependent on Vfr (Suh et al., 2002), a close homolog of the cAMP receptor protein (CRP) of *E. coli*. A metabolic signal derived from acetyl-CoA and controlling the *P. aeruginosa* T3SS might also control activity of CyaA and/or CyaB (Rietsch and Mekalanos, 2006).

Also of interest here is the fact that histidine utilization in *P. aeruginosa* interferes with production and translocation of the T3SS cytotoxic effector ExoS (Rietsch et al., 2004), indicated by the observation that a transposon insertion leading to loss of ExoS-dependent cytotoxicity causes over-expression of several histidine utilization (*hut*) genes. Rietsch and colleagues showed that the loss of cytotoxicity could be suppressed by excluding histidine from the medium and by deletion of several *hut* genes. Another suppressing mutation was localized in *cbrA*, encoding the sensor of a two-component system involved in sensing of- and responding to carbon-nitrogen imbalance. Such an imbalance might result from excessive histidine catabolism. Tryptophan catabolism also interferes with type III secretion in *P. aeruginosa* (Shen et al., 2008). This linkage might be explained by the role of tryptophan as a precursor of quorum sensing-like signaling molecules, given that expression of the *P. aeruginosa* T3SS genes is cell density-dependent.

Collectively, several studies suggest that virulence gene expression and the nutritional situation of *P. aeruginosa* are coordinated during infection. It is interesting to note that the broad metabolic adaptation manifested by *P. aeruginosa* associated with long-term colonization of cystic fibrosis (CF) patients is accompanied by a down-regulation of ExoS production (Hogardt et al., 2007; Hoboth et al., 2009). In the light of the interdependence of metabolism and virulence, down-regulation of ExoS in this case might be explained by metabolic adjustments that lead to altered regulation of T3SS gene expression. In line with this explanation, ExoS down-regulation in late CF isolates can be overcome by overproduction of the T3SS transcriptional activator ExsA (Dacheux et al., 2001a; Hoboth et al., 2009). Finally, since *P. aeruginosa* T3SS is apparently designed to provide nutrients to the bacteria, coordination between metabolic states and expression of virulence factor genes is intuitive.

### *Salmonella*: T3SS-mediated recruitment of nutrients for my brother

**Figure 2:**
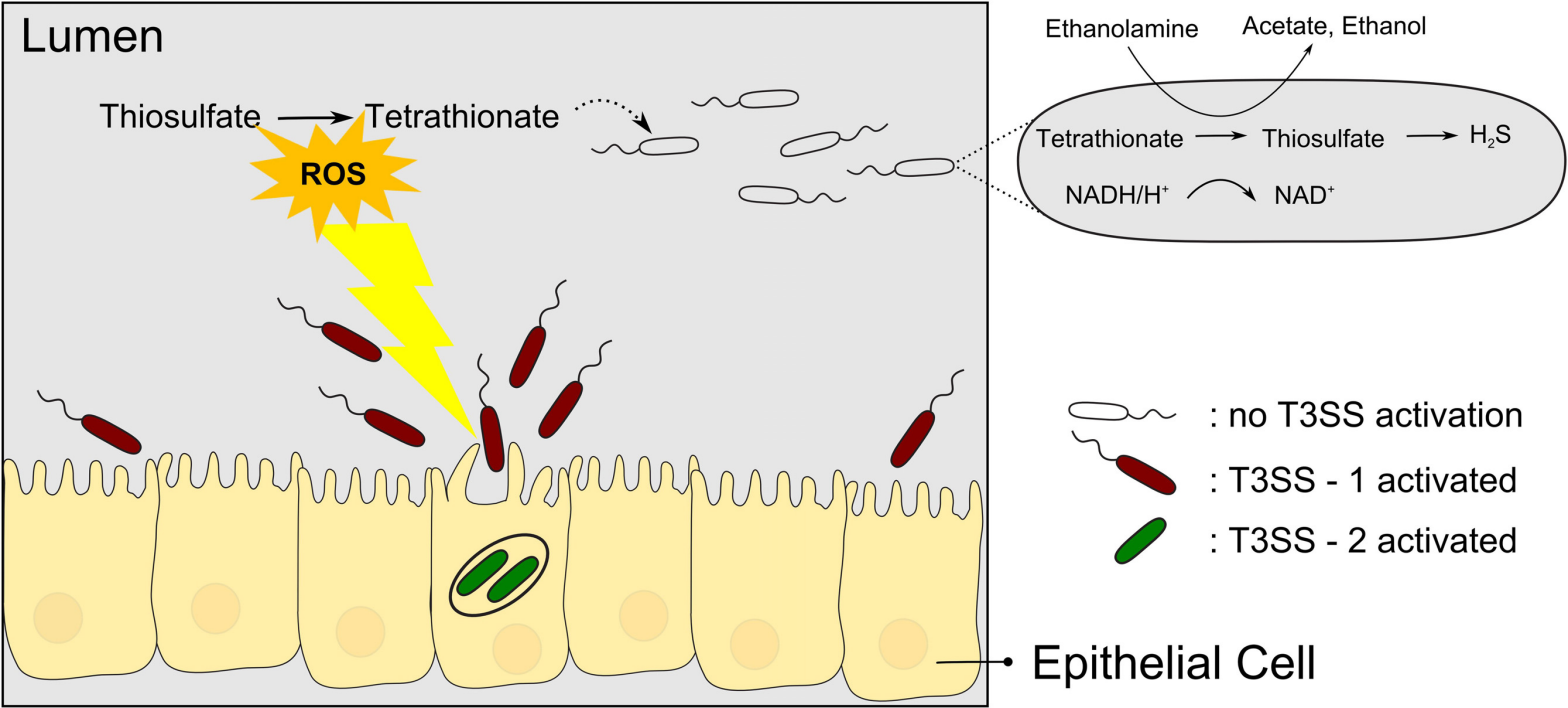
**Salmonellae recruit new nutrients for their brothers by means of self-destructive deployment of a T3SS**. T3SS-1 mediated invasion of the gut tissue is mostly a kamikaze mission but the inflammatory response induced comprises the release of reactive oxygen species (ROS) which results in the formation of tetrathionate, an alternative electron acceptor. Surviving salmonellae can then use tetrathionate to metabolize ethanolamine to proliferate and outcompete the normal gut flora.

Tetrathionate has long been used as a supplement to media for enrichment of *Salmonella* (Muller, 1923). However, only in recent years has the biological context of tetrathionate usage by *Salmonella* been elucidated in more detail, revealing an intricate relation to T3SS-based virulence. *Salmonella* species causing typhoid fever and gastroenteritis deploy two T3SSs encoded in *Salmonella* pathogenicity islands SPI-1 and SPI-2, here termed T3SS-1 and T3SS-2, respectively. With reference to the link between pathogenicity and metabolism, a sub-population of *Salmonella* Typhimurium seems to use the T3SS-1 altruistically but self-destructive to the nutritional benefit of the surviving subpopulation (Ackermann et al., 2008). During colonization of the mouse gut, only a subpopulation of *S*. Typhimurium expresses the T3SS-1 genes (15% of the salmonellae found in the lumen), which then invades the gut tissue and triggers an inflammatory response. While the invading bacteria seem to be killed, the subpopulation residing in the gut lumen is enabled to compete with the normal gut flora and presumably to acquire the released nutrients. Specifically, Winter et al. (2010) could show that the respiratory burst induced by the T3SS-1 results in formation of the alternative respiratory electron acceptor tetrathionate in the gut. As a consequence, tetrathionate then enables salmonellae to use ethanolamine as a substrate which is abundant and provided by the host yet does not promote growth effectively by fermentation (Thiennimitr et al., 2011) (Figure 2). It is interesting to note that the *ttr* genes required for tetrathionate respiration are encoded within SPI-2 in close proximity to T3SS-2 genes (Hensel et al., 1999) although it is hitherto unknown whether there is a linkage between *ttr* genes and T3SS-2 genes beyond that co-localization within SPI-2.

It is worthwhile mentioning here that *Y. enterocolitica* also harbors the tetrathionate pathway suggesting that inflammatory responses are exploited in a similar way (Thomson et al., 2006; Winter et al., 2010).

Another layer of complexity is observable in *Salmonella* Typhimurium strains harboring the SopEΦ prophage. SopE, an effector protein translocated by T3SS-1, can induce increased levels of nitric oxide (NO) within the mouse gut, which as a net result of interaction with reactive oxygen species (ROS) can lead to increased nitrate levels, that is generation of another alternative electron acceptor that is preferred over tetrathionate and promoting growth in the gut lumen (Lopez et al., 2012).

Taken together, the *Salmonella* T3SS-1 is not only deployed to mediate entry into host cells by the manipulation of host cellular signaling but also to unlock novel nutritional resources, suggesting intimate cross-talk between the physiological state and the expression of virulence factor genes. In fact, the central regulator gene of the *Salmonella* T3SS-1, *hilA*, is activated by carbon source deprivation and (p)ppGpp (Pizarro-Cerda and Tedin, 2004; Song et al., 2004). Moreover, *hilA* and many other SPI-1 encoded genes are controlled by metabolic enzyme genes, *adhE*, encoding a bifunctional acetaldehyde-CoA/alcohol dehydrogenase and *pflB*, encoding pyruvate formate lyase I, of the pyruvate metabolism (Abernathy et al., 2013). Furthermore, expression of several virulence factor genes including those of T3SS–1, is controlled by short-chain fatty acids that might serve as nutrients in the intestine (El-Gedaily et al., 1997; Utley et al., 1998; Lawhon et al., 2002). The DNA-binding protein Fis might play a decisive role in coordinating metabolism and virulence in *Salmonella* (Kelly et al., 2004), since it controls expression of genes in several pathogenicity islands on the one hand and of genes encoding metabolic pathway components involved in fatty acid and acetate metabolism including the glyoxylate shunt on the other. The role of the glyoxylate shunt during infection in particular is unclear since it seems to depend on infection stages and differs between *Salmonella* serovars (Utley et al., 1998; Faucher et al., 2006; Tchawa Yimga et al., 2006; Eisenreich et al., 2010; Bowden et al., 2014). In *Salmonella* central metabolic enzymes including key enzymes of glycolysis, gluconeogenesis, TCA cycle and glyoxylate shunt are extensively acetylated to control metabolic fluxes, imposing yet another layer of complexity in understanding regulatory networks (Wang et al., 2010).

Very recently, another striking example of a direct interrelationship between a T3SS and metabolism was discovered in *Salmonella* (Maze et al., 2014). The phosphotransferase system (PTS) of bacteria catalyses the uptake and concomitant phosphorylation of sugars using phosphoenolpyruvate (PEP) as donor of the phosphoryl group (Deutscher et al., 2006). Now, the PTS component EIIA^Glc^ was shown to associate with the SPI-2 encoded T3SS-2 (Maze et al., 2014). Moreover, effector secretion by the T3SS-2 required EIIA^Glc^ and systemic *Salmonella* virulence was found to critically depend on EIIA^Glc^. Interestingly, however, the phosphorylation-dependent sugar transport function of EIIA^Glc^ and its regulatory impact on adenylate cyclase activity did not account for the phenotype. Rather, a direct interaction between EIIA^Glc^ and T3SS-2 was suggested (Maze et al., 2014). It remains to be determined whether this moonlighting function of EIIA^Glc^ is of purely structural nature or whether it contributes to a coordination of metabolic and virulence functions.

All in all, the literature on *Salmonella* contains numerous arguments of a close interrelation between metabolism and virulence, notably referring to the T3SSs.

### Further examples

The examples presented and discussed above are systems in which the link between metabolism and T3SS has been most extensively studied. Interested readers will find further examples and hints in studies on the *Aeromonas* T3SS. The regulatory interplay between the pyruvate dehydrogenase complex and T3SS regulator genes *aexT* and *aopN* in *A. hydrophila* reported by Vilches et al. (2009) is strongly reminiscent of the interrelation between *aceAB* and T3SS in *P. aeruginosa* discovered by Dacheux et al. (2002) and discussed above.

In another example, enteropathogenic *E. coli* (EPEC) secrete glyceraldehyde-3-phosphate dehydrogenase (GAPDH) via a T3SS (Kenny and Finlay, 1995). GAPDH might be injected into the host cell cytosol and interfere with host cellular metabolism e.g., by inhibiting host cellular GAPDH complexes through formation of unproductive heterooligomers or by increasing fluxes within the glycolytic pathway. Alternatively, GAPDH has a moonlighting function independent of its enzymatic activity, fulfilling in bacteria and/or the host. For example, GAPDH is also secreted by different *Streptococcus* species, and exhibits ADP-ribosylating activity and immunomodulatory properties (Pancholi and Fischetti, 1992, 1993; Madureira et al., 2007). It is interesting to note in this context, that a T3SS effector, NleB, found in EPEC and EHEC (enterohaemorrhagic *E. coli*), was recently found to act as glycosyltransferase toward host cellular GAPDH to inhibit NF-κB activation, illustrating yet another moonlighting facet of GAPDH (Gao et al., 2013). It remains to be determined whether it is just by chance that EPEC secrete and possibly translocate GAPDH into host cells in which they also target host cellular GAPDH and whether these phenomena are first indications of an interlock of metabolism and T3SS on both the bacterial and the host cellular side.

Finally, AdhE, a bifunctional acetaldehyde-CoA dehydrogenase/alcohol dehydrogenase was shown to control expression of the EHEC T3SS (Beckham et al., 2014), a finding reminiscent of the relation between *adhE* and SPI-1 found in *Salmonella* (Baumler et al., 1994; Abernathy et al., 2013).

## Conclusions and perspectives

### A novel interrelationship between virulence functions and metabolism

In very general terms, the environment within the host determines the physiological state of the pathogen, which in turn governs production and functioning of virulence factors. Our knowledge of the complexity of these regulatory networks is further increasing (as illustrated above; see Table 1 for summary). Our way of looking at bacterial pathogenesis is still dominated by the question of which virulence factors are produced and how metabolism controls virulence gene expression. However, virulence factors conversely control metabolic functions in a coordinated manner. Furthermore, there is evidence that virulence functions are deployed to manipulate host metabolism, potentially for the benefit of the metabolism in the pathogen. Clearly, we face a novel interrelationship between virulence and metabolic functions. However, do such phenomena reflect the exception or the rule?

**Table 1:** **Summary of the metabolic pathways impacted by T3SSs and/or vice versa in the order of appearance in this manuscript**.

**Species**	**T3SS component**	**Gene/Pathway**	**References**
*Yersinia*	LcrQ/YscM	*ppc*/PEP carboxylase	Schmid et al., 2009
	*lcrG*	*aspA*/aspartase	Du et al., 2009
	pCD1	β–oxidation, glyoxylate shunt	Moncla et al., 1983
	*lcrG*	*aceA, aceB*/glyoxylate shunt	Du et al., 2009
	*sycO*-*ypkA*-*yopJ*	cAMP-CRP	Zhan et al., 2009
*Pseudomonas*	T3SS	*aceAB*/pyruvate dehydrogenase	Dacheux et al., 2002
	T3SS	*cyaA,cyaB*/cAMP-Vfr	Wolfgang et al., 2003
	ExoS	*hut*/histidine utilization	Rietsch et al., 2004
	T3SS	*kynA*/tryptophan metabolism	Shen et al., 2008
*Salmonella*	T3SS-1 (SPI-1) and SPI-2	*ttr*/tetrathionate respiration	Hensel et al., 1999; Winter et al., 2010
	SopE (T3SS-1)	NO, nitrate respiration	Lopez et al., 2012
	*hilA* (SPI-1)	(p)ppGpp	Pizarro-Cerda and Tedin, 2004; Song et al., 2004
	T3SS-1 (SPI-1)	*adhE, pflB*/pyruvate metabolism	Abernathy et al., 2013
	T3SS-1 (SPI-1)	Short-chain fatty acids	El-Gedaily et al., 1997
	T3SS-2 (SPI-2)	*crr*/PTS component EIIA^Glc^	Maze et al., 2014
*Aeromonas*	*aexT, aopN*	*aceA*/pyruvate dehydrogenase	Vilches et al., 2009
*E. coli* (EPEC)	T3SS	Secretion of glycolytic enzyme GAPDH	Kenny and Finlay, 1995
*E. coli* (EHEC)	T3SS	*adhE*/pyruvate metabolism	Beckham et al., 2014

The multiple convergent evolution of dense regulatory networks to coordinate virulence and metabolic functions suggests strong selection pressures acting and indicates that such cross-talk could be the rule rather than the exception. From a cybernetic point of view, many regulator circuits seem plausible.

### Cross-talk via anaplerotic functions: a common theme

From the point of view of the invader, infection usually means unbalanced diet and shortage. From *in vitro* studies it is known that growing on a limited number of carbon sources requires considerable reprogramming of metabolic fluxes via anaplerotic enzymes. From the above examples, it seems as if such anaplerotic enzymes are preferred nodal points for communication with virulence functions, lending support to the view that “unbalanced diet” is a condition frequently faced by pathogens.

*Agrobacterium tumefaciens* induces crown gall tumors on its plant hosts by means of a type 4 secretion system (T4SS). The virulence (*vir*) genes are localized on the Ti (tumor-inducing) virulence plasmid and are controlled by the two-component system VirAG. Deletion of *pckA* encoding the anaplerotic enzyme phosphoenolpyruvate carboxykinase (PckA) attenuates virulence through reduced *vir* gene expression (Liu et al., 2005) illustrating that such regulatory circuits are not restricted to pathogens running T3SSs.

This view is further underscored by the fact that anaplerotic reactions play a key role during infection in many pathogens with less well-defined armament. A crucial function of the glyoxylate shunt during infection, for example, has been demonstrated for *Mycobacterium tuberculosis* (McKinney et al., 2000) and for the fungal pathogen *Candida albicans* (Lorenz and Fink, 2001).

### Metabolism of the host

For reasons of clarity and scope, this review was confined mainly to the central carbon metabolism of bacterial pathogens and its interplay with virulence functions related to T3SSs. Availability of all kind of nutrients (not only of carbon), however, depends on host metabolism, which differs depending on localization within tissues and cells and, moreover, is not static but changes over the course of infection. As an example, sepsis is accompanied by a massive redistribution of carbon and nitrogen fluxes within the infected individual, accompanied by decreased glutamine uptake in the intestine and a switch from glutamine uptake state to release in the kidney. In contrast, proliferating lymphocytes increase glutamine utilization upon sepsis and the liver becomes the major glutamine consumer under these conditions (Karinch et al., 2001). Consequently, many pathogens will have to adapt their metabolism to rearrangements of the host metabolism.

Changes of host metabolism in the context of infection can be viewed as a host response or as directed manipulation of host metabolism by the pathogen. As discussed above, there are hints that virulence factors are deployed to manipulate host metabolism and to serve the release of nutrients. The function of virulence factors is often discussed in a biased way, neglecting that the primary goal of the pathogen is not to fool the host but to replicate in it. We expect that manipulating host metabolism, on the single host cell level as well as on the tissue, organ and systemic level will turn out to be a major issue of pathogens.

### Systematic investigation of cross-talk between virulence and metabolism: toward a systemic view of infection

Much of the above discussion is based on incidental observations. So, how should we examine cross-talk between metabolism and virulence functions more systematically? As a first step, literature from the pioneers studying pathogens may still store many valuable hints to follow-up. Further, it is important to screen the transcriptomics, proteomics and metabolomics datasets to identify novel interrelationships. Clearly, a comprehensive view will require other “omics” such as “interactomics” and “fluxosomics,” including data on the host. Integration of these data to form a penetrable view of infection will be a major intellectual challenge. Feeding systems biologists with “omics” data is a must to drive modeling toward a systemic view of infection. It is however clear that applicability of modern systems biology approaches is still limited from an economic and data handling perspective but also with regard to sensitivity as well as temporal and spatial resolution. For example, bacterial pathogens practice division of labor as do cells of their hosts so that the different events on which we sum up in most experiments cannot be resolved and the results misguide us. Moreover, type three secretion is not only a very complex but also a highly dynamic process which poses a specific challenge to the temporal resolution of our methodology. Single cell analysis is now rapidly developing on the “omics” level and at least DNA and RNA sequencing will become broadly available (Pan, 2014) to resolve some of the heterogeneity that we are faced with.

In conclusion, cohesive use of physiology and molecular biology is needed to enlighten the complex events of infection: we cannot understand infection without understanding of metabolism of host and the pathogen.

### Conflict of interest statement

The authors declare that the research was conducted in the absence of any commercial or financial relationships that could be construed as a potential conflict of interest.
